# A Case of Pulmonary Alveolar Proteinosis in a 15-Year-Old Female Patient

**DOI:** 10.7759/cureus.39254

**Published:** 2023-05-20

**Authors:** Omar R Khalil, Osama S Matar, Mohammad H Abed Alhaleem, Ann A Attili, Suhib M Ibrahim

**Affiliations:** 1 Internal Medicine, Al-Quds Univeristy, Jerusalem, PSE; 2 Internal Medicine, Al-Quds University, Jerusalem, PSE; 3 Internal Medicine, An-Najah National University, Nablus, PSE

**Keywords:** whole lung lavage, bronchoalveolar lavage, high-resolution ct scan, interstitial lung disease, pulmonary alveolar proteinosis

## Abstract

Pulmonary alveolar proteinosis (PAP) is an extremely rare pulmonary disease that can be classified into primary, secondary, or congenital types. It typically presents with a pattern of interstitial lung disease. This rare condition is even rare in the adolescent or pediatric age group, making this case particularly rare and interesting.

We report a case of a 15-year-old girl who presented with a four-month history of dry cough and exertional dyspnea. After performing a high-resolution computed tomography (HRCT) scan and bronchoalveolar lavage (BAL) with analysis of the BAL fluid, she was eventually diagnosed with PAP. She was then referred to a higher qualified center, where a whole lung lavage (WLL) was performed, resulting in significant improvement of her symptoms.

## Introduction

Pulmonary alveolar proteinosis (PAP) is a very rare disease in which there is an accumulation of alveolar surfactant and its components within the alveoli [1]. It is classified into primary, secondary, and congenital according to its pathophysiology and etiology [1]. Primary PAP is caused by a disruption in the granulocyte-macrophage colony-stimulating factor (GM-CSF) signaling; the autoimmune type is characterized by the presence of autoantibodies against GM-CSF, and it represents 90% of all PAP cases. The hereditary type happens as a result of GM-CSF receptor gene mutations [1,2]. Secondary PAP results from different underlying diseases that affect the number of alveolar macrophages, and also cause dysfunction in their clearance activity. Various diseases are involved, including bacterial, viral, or fungi infections, neoplasms like lymphomas, inorganic dust exposure, and immunodeficiencies. Overall, it represents 5%-10% of all PAP [2-4]. Congenital PAP is characterized by mutations in surfactant protein genes which, as a result, affect the production and the physiological role of surfactant. And it represents around 2% of all PAP [1,3].

The incidence of PAP is 0.5 to 1.5 cases per one million. It is mainly seen in males, with a male-to-female ratio of 2:1. The mean age of patients is between 30-50 years old, and many of these patients are either current or previous smokers; around 80% of them have a previous history of smoking [4]. One-third of patients with PAP are asymptomatic, and if they present with symptoms then they are mostly non-specific. Dyspnea is most commonly reported, then comes cough and fatigue, and weight loss [1]. In secondary PAP, the most commonly reported symptom is fever, then comes dyspnea with exertion and cough. Physical examination is mostly unremarkable, but crackles and clubbing have been reported [5].

## Case presentation

**Figure 1:**
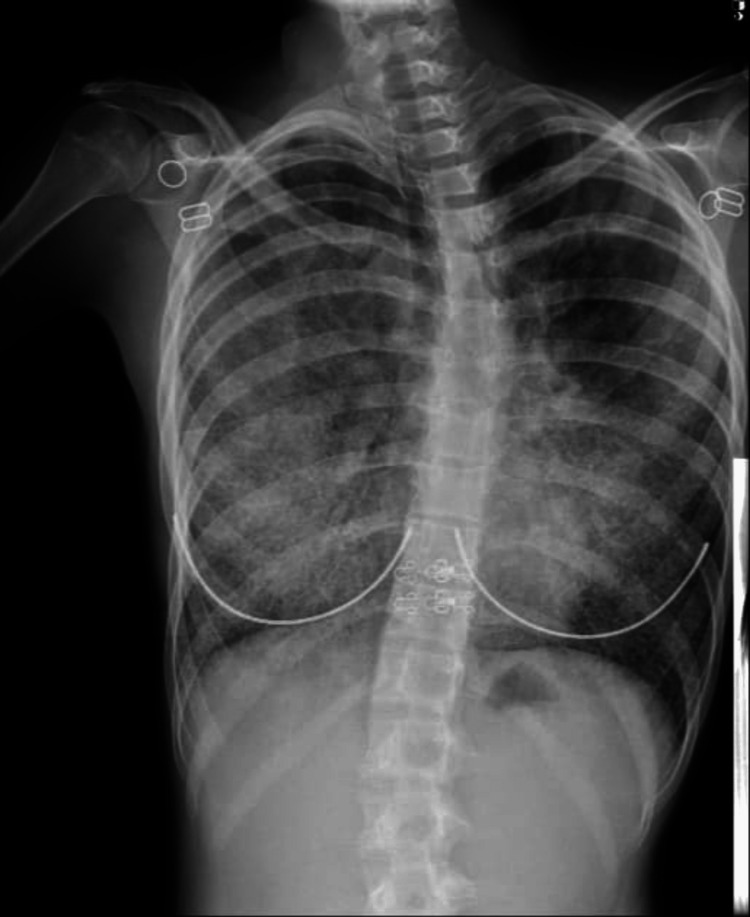
Chest x-ray at presentation with bilateral infiltrates.

A 15-year-old female patient, with no significant medical history except for COVID-19 infection one year prior and tonsillectomy, presented to the emergency department (ED) with a one-week history of fever reaching 39℃. She complained of chills, runny nose, cough, shortness of breath, right-sided chest pain on inspiration, and general fatigue. The patient also reported a four-month history of paroxysmal episodes of dry cough, associated with runny nose, sneezing, and headaches, and a month of shortness of breath upon walking 400m on flat ground, despite multiple antibiotic regimens with no significant improvement. The patient denied having any orthopnea, paroxysmal nocturnal dyspnea, limb edema, weight loss, night sweats, rashes, or other symptoms suggestive of connective tissue disease. There was no exposure to allergens, dust, or any other environmental factors. Physical examination revealed a dyspneic patient with oxygen saturation of 90% on room air and bilateral basal crepitations on the chest. CBC showed white blood cells (WBC) 14.000/mm^3^, and a chest radiograph (x-ray) revealed bilateral alveolar infiltrates more predominant on the right side (Figure 1). The rapid AG test for COVID-19 was negative. The patient was admitted to the hospital for one week and treated with IV antibiotics for bronchopneumonia.

**Figure 2:**
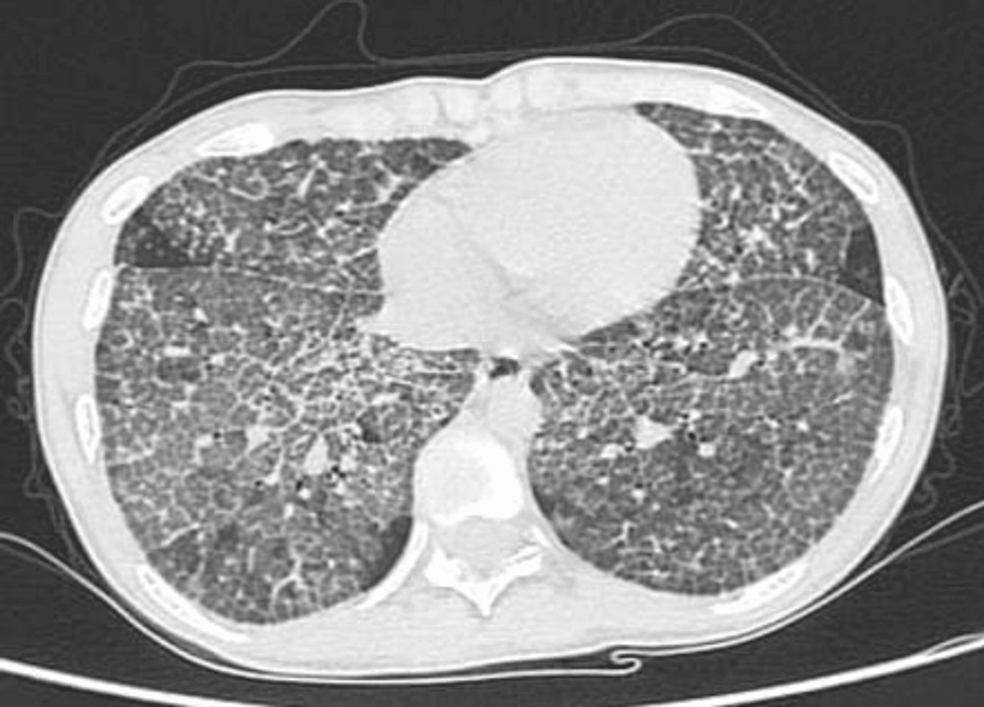
High resolution chest CT scan with crazy-paving appearance.

During the hospitalization period, a high-resolution chest CT (Figure 2) was performed, which showed diffuse multi-lobar smooth thickening of inter-lobar and intra-lobar septal lines, and ground glass opacities with a crazy-paving appearance, findings consistent with PAP. Other laboratory investigations, including arterial blood gases, showed a PaO_2_ of 66.2 mmHg, PaCO_2_ of 33.1 mmHg, and pH of 7.41. A pulmonologist was consulted and recommended doing ANA, ENA profile, RF, ANTI-CCP, HIV, and hepatitis profile, which all came back negative. The patient was then referred for bronchoscopy and sequential bronchoalveolar lavage (BAL) to confirm the diagnosis of PAP. Bronchoscopy revealed normal vocal cords, a normally shaped trachea, a sharp carina, and patent airways with no endobronchial lesions. Sequential BAL from the right lower lobe showed a milky white return that became denser with each lavage. Sections of the biopsy showed small fragments of bronchial mucosa with non-specific reactive inflammatory infiltrates. PAS stain was positive, and the granular material features were consistent with alveolar proteinosis. Pulmonary function tests revealed FVC: 3.33 L (66% of predicted), FEV1 2.83 L (73%), FEV1/FVC 113%, TLC 4.41 L (50%), and DLCO 8.10 mmoL/min/kPa (28%).

Given the patient's history and age, and absence of well-defined cause of secondary PAP, the most likely diagnosis in our patient is primary PAP and most likely autoimmune PAP. Unfortunately, the confirmatory test (anti-GM-CSF antibodies) was not available to confirm that diagnosis. The patient was then referred to another facility to undergo therapeutic whole lung lavage (WLL), as the procedure was not available in our hospital, which resulted in an improvement in symptoms as noticed in her follow-up visit two weeks after the lavage. Unfortunately, we were unable to obtain a radiological study of the patient after the procedure from the center.

## Discussion

PAP is typically diagnosed in adults between the ages of 40 and 50 [6]. PAP is a rare disease in children and has an estimated occurrence of two cases per million in those under the age of 18 [7]. Approximately 30% of those diagnosed with PAP are asymptomatic, while the rest develop non-specific complaints. The most commonly reported symptom is dyspnea, especially upon exertion, followed by cough with or without white sputum and systemic signs such as fever and weight loss [3,6]. Our patient presented early at the age of 15 with fever, shortness of breath, and chest pain, which are usually present in cases of a superimposed infection and have been rarely reported as the first manifestation [1]. The disease had an insidious onset, with dry cough being the predominant complaint for four months prior to her ER visit. Three months later, the patient began experiencing exertional dyspnea after walking a distance of approximately 400m. She had tried multiple antibiotic regimens during that time with no benefit.

Diagnosis of PAP is made with proper complete history taking, physical examination, radiological findings, BAL cytology with possible biopsy, and biomarkers. Radiological findings are typically symmetrical opacities in the mid and lower lung field bilaterally. Ground-glass opacities are frequently seen in autoimmune and hereditary PAP on high-resolution computed tomography (HRCT) [5]. If PAP is suspected after history, physical examination, and radiological findings, going for early anti-GM-CSF autoantibodies is found useful to make a diagnosis of the most common type of PAP, autoimmune PAP. With this being followed, multiple invasive procedures could be minimized as secondary PAP requires multiple procedures to be diagnosed [5]. The fluid of BAL shows a thick, turbid appearance, and milky sediment. Moreover, the cytology of this fluid with periodic acid Schiff (PAS) stain and oil-red-O stain shows foamy macrophages and dirty sediment [4]. The combination of radiology and BAL cytology makes the choice of performing a lung biopsy unnecessary. An HRCT scan was ordered for our patient and showed a ground glass appearance with smooth thickening of interlobular and intralobular septal lines resulting in a so-called crazy-paving appearance; findings strongly suggest PAP [3]. To confirm the diagnosis, a BAL was done and showed a milky fluid appearance, and a PAS-positive material, which is typically seen in other reported cases [8]. However, anti-GM-CSF autoantibodies were not available in our hospital or the referral center, so we couldn't exactly determine the exact type in our case.

The primary treatment for PAP is WLL. In cases of secondary PAP, treating the underlying cause is crucial. Congenital PAP is mainly managed with supportive therapy, although lung transplantation has been reported to have successful outcomes [4,5]. In older children, treatment for PAP may involve WLL, which has been reported to improve symptoms in some case reports [9]. This therapy was considered for our patient, and positive outcomes were observed following the procedure. Her pulmonary function tests showed improvement compared to previous readings, and her symptoms were alleviated, further supporting the efficacy of WLL in pediatric PAP cases.

## Conclusions

In conclusion, PAP should be considered in the differential diagnosis for patients presenting with persistent dry cough and exertional dyspnea, regardless of age. An HRCT scan of the chest is an essential initial evaluation when PAP is suspected based on a patient's history. Confirmatory tests such as BAL cytology or lung biopsy may be necessary to establish a definitive diagnosis.

In specific cases, it is crucial to differentiate between the various types of PAP, particularly the autoimmune subtype. Autoantibody testing plays a vital role in identifying autoimmune PAP, and whenever possible, it should be pursued as part of the diagnostic workup. However, it is important to acknowledge that access to autoantibody testing may be limited in certain regions or healthcare settings. In such circumstances, a systematic approach to ruling out other types of PAP, including congenital and secondary forms, can be an acceptable method to diagnose autoimmune PAP. Although this approach may not provide definitive confirmation, it helps guide appropriate management decisions when specific testing is unavailable or impractical.
